# IMPACT OF REAL-LIFE ENVIRONMENTAL EXPOSURES ON REPRODUCTION: Systemic and ovarian impacts of heat stress in the porcine model

**DOI:** 10.1530/REP-24-0217

**Published:** 2024-11-11

**Authors:** Aileen F Keating, Jason W Ross, Lance H Baumgard

**Affiliations:** 1 Department of Animal Science, Iowa State University, Ames, Iowa, USA

## Abstract

**In brief:**

This review describes how heat stress causes systemic endocrine and metabolic alterations that contribute to intracellular ovarian perturbations, resulting in female infertility.

**Abstract:**

Heat stress (HS) in mammals results from an imbalance in heat accumulation and dissipation. Fertility impairments consequential to HS have been recognized for decades in production animals, and more recently, observations have been extended to other species, including women. There are several systemic impacts of HS that can independently affect reproduction, including metabolic endotoxemia, reduced plane of nutrition, and endocrine disruption. At the level of the ovary, molecular pathways are altered by HS, such as inflammation, JAK–STAT, PI3K, oxidative stress, cell death, and heat shock response. Taken together, impaired ovarian function contributes to seasonal infertility that results from HS. This review paper describes the physiological and endocrine systemic impacts of HS that may independently and collaboratively impair fertility in the porcine model. The review then details ovarian intracellular events that are altered during HS and finally determines future needs in this area of research.

## Introduction

The hottest global environmental ambient temperatures have occurred in recent decades (Papalexiou *et al.* 2018, Ebi *et al.* 2021), which can impact human and animal health. Recently, ambient temperatures have steadily increased by 0.7°C per decade and are expected to rise from 1 to 2°C by 2050 (Costello *et al.* 2009). Extreme weather conditions, including severe heat events, are one consequence of this climate variability (Thornton *et al.* 2014). Increased environmental temperatures threaten human and animal health due to increased incidence of infectious and vector-borne diseases (McMichael & Kovats 2000), malnutrition and reduced nutrient absorption (Costello *et al.* 2009), elevations in respiratory and cardiovascular illnesses (Robine *et al.* 2008), and increased morbidity and mortality (Kovats & Kristie 2006, Robine *et al.* 2008, Costello *et al.* 2009).

Heat stress (HS) occurs when environmental conditions (ambient temperature, relative humidity, and solar radiation) coupled with endogenous heat production generate a thermal condition culminating in increased core body temperature (Bernabucci *et al.* 2010). There are socio-economic factors to also consider regarding the impacts of climate change and particularly HS, since poverty can reduce access to air conditioning, coupled with the incidence of urban heat islands in poorer neighborhoods, where asphalt and concrete re-radiate solar energy (Memon *et al.* 2008, Adams *et al.* 2023, Burlotos *et al.* 2023, Cleland *et al.* 2023). In agricultural animal production, HS has been documented for decades to impair lactation, reproduction, growth, and body composition (Baumgard & Rhoads 2013). Regarding female reproduction, HS causes infertility and poor pregnancy outcomes, anovulation, reduces conception rates, and lowers pregnancy maintenance (Love 1978, Love *et al.* 1993, Xue *et al.* 1994) and in North America, swine reproductive efficiency is compromised during the summer months and early autumn (Ross *et al.* 2017). In the below sections, systemic physiological changes that are plausible contributors to HS-induced infertility will be described. In addition, the links between some of these changes are provided; however, the temporal pattern of change, and thus the order of timing of the HS that ensues, remains undescribed in the literature. The ovarian intracellular pathways that are documented to change in the heat-stressed pig are also described. This review focuses on the impacts of HS in the pig as an agricultural and biomedical model (Lunney *et al.* 2021, Bergen 2022, Hou *et al.* 2022, Lu *et al.* 2022), but it should be noted that the impacts of HS in other species related to their fertility outcomes have been described in the literature but are outside the scope of this review.

### Heat stress negatively impacts female reproduction

Reproductive alterations attributed to HS in pigs include anovulation, reduced conception rates, and compromised pregnancy (Love 1978, Love *et al.* 1993, Xue *et al.* 1994). In cattle, *in vitro* exposure of preovulatory follicles to HS decreased E_2_ in granulosa cells (Roth *et al.* 2001, Bridges *et al.* 2005), reduced oocyte competence (Zeron *et al.* 2001, Al-Katanani *et al.* 2002), and reduced fertility outcomes (Al-Katanani *et al.* 1999, Roth 2021).

Additionally, embryonic development is impaired (Ealy *et al.* 1993, Edwards 1997), and fetal growth is reduced (Collier *et al.* 1982, Wolfenson *et al.* 1988, Thureen *et al.* 1992) in cattle, sheep, and goats by HS. *In utero* HS results in increased core body temperature postnatally in swine along with increased carcass lipid deposition and reduced size of the liver, heart, and kidney (Johnson *et al.* 2013, 2015). Similar effects of gestational HS on future body temperature indices and productivity have been reported in ruminants (Ahmed *et al.* 2017).

In women, increased ambient temperatures are associated with reduced antral follicle counts (Gaskins *et al.* 2021) and stillbirth (Nyadanu *et al.* 2022). Interestingly, HS tolerance in female soldiers is related to their menstrual cycle stage, with improved HS tolerance observed during the luteal phase (Yanovich *et al.* 2019). Pregnancy is a sensitive developmental stage for HS effects (Wyrwoll 2023) as hyperthermia increases the risk of miscarriage (Rekha *et al.* 2024) and maternal hypertension (Mao *et al.* 2023). A higher risk of pre-term birth is also supported by heat events, as evidenced by the analysis of birth data following the 1995 heat wave in Chicago, in which an association between pre-term birth and hyperthermia was supported and racial disparity was demonstrated (Gordon *et al.* 2023).

**Figure 1:**
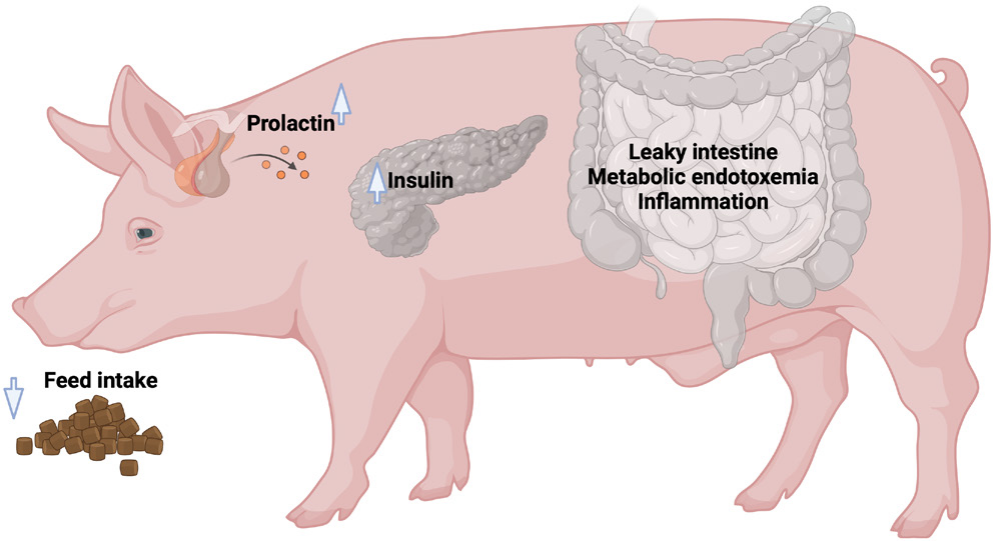
Systemic HS-induced perturbations. During HS, several systemic alterations can contribute to ovarian dysfunction independent of hyperthermia. These include hypophagia, hyperprolactinemia, hyperinsulinemia, impaired intestinal integrity, and inflammation.

Before considering the direct effects of HS on the reproductive tract, systemic impacts will first be introduced in this review, which can themselves indirectly cause reproductive dysfunction and are plausible contributors to HS-induced infertility. These include reduced plane of nutrition, leaky intestine, systemic inflammation, and endocrine disruption, as illustrated in Fig. 1.

#### Reduced plane of nutrition

Heat stress causes inappetence (Baumgard & Rhoads 2013) and malnutrition can also cause infertility (Were *et al.* 2020, Salem 2021, Sun *et al.* 2021). Caloric restriction alters fertility (Costermans *et al.* 2020, Sun *et al.* 2021), and ovarian reserve (Garcia *et al.* 2019, Zanini *et al.* 2020). Metabolic hormones, such as leptin and ghrelin, partially regulate puberty onset and female fertility (Athar *et al.* 2024), and a reduced plane of nutrition delays puberty onset in humans (Muñoz & Argente 2002, Muñoz-Calvo & Argente 2016) and animals (Byrne *et al.* 2018, Heslin *et al.* 2020, Bruinjé *et al.* 2021). It was estimated that reduced energy intake in HS cows could account for ~50% of reduced lactational performance (Rhoads *et al.* 2009); however, the impact of a reduced plane of nutrition on reproductive performance during HS is not known but is worthy of consideration during HS-induced infertility.

The serum metabolome of pre-pubertal HS pigs was altered (Roach *et al.* 2024*b*) such that aminomalonic acid, involved in the response to protein oxidative stress, was reduced. Decreased serum campesterol due to HS was also noted, which may contribute to endocrine disruption since campesterol has the same core structure as cholesterol with different side chains and competes with cholesterol following intestinal absorption (Choudhary & Tran 2011). Hydroxylamine was increased by HS and has mutagenic properties, raising concern about its elevation by hyperthermia (Gross 1985). Amino acid alterations have also been reported in cumulus–oocyte complexes subjected to HS (Kawano *et al.* 2024, Mzedawee *et al.* 2024). Thus, metabolomic consequences of heat are detectable following a thermal load.

#### Intestinal hyperpermeability

During HS, blood is redistributed to the periphery to enhance heat dissipation (Lambert *et al.* 2002), causing the gastrointestinal tract blood supply to constrict in order to maintain systemic blood pressure (Rowell *et al.* 1968, Deschamps & Magder 1994, Mayorga *et al.* 2018). Reduced intestinal integrity, a condition colloquially referred to as ‘leaky gut’, ensues since enterocytes are extremely sensitive to oxygen and nutrient restriction (Glover *et al.* 2016). A leaky intestine results in the efflux of intestinal contents into the local, portal, and systemic circulation (Amar *et al.* 2008, Al-Attas *et al.* 2009, Hawkesworth *et al.* 2013), and the microorganisms inhabiting the intestinal tract can thereby be introduced to several organs; a scenario resulting in endotoxemia (Cani *et al.* 2007, Luche *et al.* 2013). HS-induced intestinal hyperpermeability has been repeatedly demonstrated in rodents, farm animals, dogs, cats, and humans (Lian *et al.* 2020, Garcia *et al.* 2022).

The Gram-negative bacterial cell wall component (Rietschel *et al.* 1994), lipopolysaccharide (LPS), is one component that infiltrates into the systemic circulation causing metabolic endotoxemia (Cani *et al.* 2007, Luche *et al.* 2013). There are myriad reproductive and systemic effects of lipopolysaccharide (LPS) (reviewed in Bidne *et al.* (2018*a*)); one of which is the inflammatory response mediated through the toll-like receptor 4 (TLR-4) pathway (Loppnow *et al.* 1989). Bovine ovarian cortical strips that are enriched in primordial follicles were exposed to LPS *in vitro* and had reduced the primordial follicle pool and increased cellular atresia. Further support for TLR4 functionality in ovarian dysfunction is evidenced through depletion of the ovarian reserve that occurs in wildtype mice due to LPS exposure but the phenotype is absent in *Tlr4*-deficient mice (Bromfield & Sheldon 2013). Also, LPS exposure reduces follicular fluid E_2_ concentrations (Magata *et al.* 2014), inhibits LH release from the anterior pituitary (Herman *et al.* 2010), reduces CYP19 abundance (Herath *et al.* 2007), and increases ovarian TLR4 protein abundance (Bidne *et al.* 2018*b*). Thus, a compromised intestinal barrier is an important consideration for the reproductive effects of HS.

In estrus synchronized post-pubertal pigs, LPS exposure for the duration of the follicular phase increased circulating insulin and elevated LPS binding protein (LBP), serum 17β-estradiol (E_2_), and hyperglycemia (Bidne *et al.* 2018*b*). Ovarian TLR4 was also increased, demonstrating ovarian responsivity to LPS in pigs (Bidne *et al.* 2018*b*). Surprisingly, considering the immune-stimulating exposure, a febrile response was absent, and women with metabolic endotoxemia also did not have a fever. Thus, chronic low-level endotoxemia, which occurs during HS, altered ovarian function and TLR4 signaling.

### Endocrine disruption

Body temperature is controlled by the preoptic region of the anterior hypothalamus (Charkoudian & Stachenfeld 2014, 2016) and estrogenic inputs to neuronal estrogen receptors in the preoptic region of the hypothalamus may contribute to regulating heat dissipation (Rance *et al.* 2013, Zhang *et al.* 2021). Exogenous E_2_ administration increased the firing rate of heat-sensitive neurons in rat preoptic hypothalamic tissue slices (Silva & Boulant 1986) suggesting that estrogenic endocrine disruption during HS might influence core temperature.

#### Insulin

To maintain euthermia, HS females reduce feed intake to minimize the thermic effect of digestion and metabolism. Despite reduced nutrient consumption, circulating insulin is increased during HS, and this is especially apparent when compared to thermal neutral (TN) animals on a similar plane of nutrition (Itoh *et al.* 1998*a*,*b*,*c*, Wheelock *et al.* 2010, Baumgard *et al.* 2011, Pearce *et al.* 2012, 2013, 2014, Baumgard & Rhoads 2013, Sanz Fernandez *et al.* 2014, 2015, Victoria Sanz Fernandez *et al.* 2015). Both basal insulin concentration and the insulin response to a glucose tolerance test are increased during HS (Baumgard & Rhoads 2013), suggesting heightened pancreatic insulin secretion, which is especially apparent when corrected for concomitant reduced feed intake. Insulin regulates cellular bioenergetics associated with carbohydrate and lipid metabolism (Saltiel & Kahn 2001) and also influences reproductive function (Brüning *et al.* 2000, Sliwowska *et al.* 2014). Conditions in which chronic hyperinsulinemia is present often have reduced female fecundity and fertility (Sakumoto *et al.* 2010).

#### Prolactin

Prolactin (PRL), produced and secreted by lactotrophs within the anterior pituitary gland (Riddle *et al.* 1933), is increased by HS in multiple species (Smith *et al.* 1977, Schillo *et al.* 1978, Ronchi *et al.* 2001, Alamer 2011, Iguchi *et al.* 2012, Baumgard & Rhoads 2013), including pigs (Baumgard & Rhoads 2013). The precise mechanism by which HS increases PRL is unknown; however, the PRL response may include sweat production, intracellular and extracellular osmotic fluid balance (Kaufman & Mackay 1983), and water intake (Schams & Himmler 1978, Schams *et al.* 1980) and/or heat shock protein induction (Blake *et al.* 1995). The amount of water loss is dependent upon the extent or length of HS, and pigs can modify their hydration status to compensate for the loss of water.

PRL stimulates the production of E_2_ and P_4_ (McNeilly *et al.* 1982) and regulates ovarian immune cell populations (Laoharatchatathanin *et al.* 2023). Hyperprolactinemia results in infertility, amenorrhea, and reduced gonadotrophin-induced E_2_ production in antral follicles (Jonassen *et al.* 1991). In women, hyperprolactinemia inhibits GnRH, thereby altering cyclicity and ovulation (Bachelot & Binart 2007). Female *Prl^−^*^/−^ mice are infertile with irregular cyclicity (Horseman *et al.* 1997, Ormandy *et al.* 1997), increased luteal cell death, and reduced P_4_ production (Grosdemouge *et al.* 2003). Hyperprolactinemia is also associated with polycystic ovarian syndrome (PCOS), galactorrhea, halted ovulation, and infertility (Bracero & Zacur 2001). A link, therefore, between increased circulating PRL and HS-induced female infertility is plausible.

The porcine PRL receptor (PRLR) gene is located on chromosome 16 (Vincent *et al.* 1997) and a genome-wide association study associated thermotolerance in pigs with gene variants on chromosome 16 (Kim *et al.* 2018). The PRLR has long (PRLR-L) and short (PRLR-S) form splice isoforms (Kelly *et al.* 1991) and signaling via the PRLR-S is less extensive compared to the PRLR-L isoform (Ormandy *et al.* 1998). Further evidence of PRL’s role in fertility is demonstrated by a *Prlr* gene variant association with PCOS (Amin & Gragnoli 2023). As regards the HS response, cows carrying a *Prlr* mutation are more thermotolerant to high temperatures and are anecdotally referred to as SLICK cows (Dikmen *et al.* 2008, Dikmen *et al.* 2014, Porto-Neto *et al.* 2018). The binding of PRL to the PRLR activates the Janus kinase/signal transducer and activator of transcription (JAK-STAT), mitogen-activated protein kinase (MAPK), or PI3K cellular pathways. Janus kinase 2 (JAK2) dimerizes, and its autophosphorylation recruits STAT 1, 3, and 5 proteins, followed by nuclear translocation to stimulate PRL-responsive gene transcription. Some evidence that HS in pigs alters the JAK–STAT proteins has emerged (see later section).

Reduced plane of nutrition and higher circulating PRL have been documented in pre-pubertal gilts (Roach *et al.* 2024*b*), but there is an opposite response in mares (McManus & Fitzgerald 2000) and dairy cows (Lacasse & Ollier 2015), in which reduced circulating PRL was noted during feed restriction. Potentially related to the observation of reduced feed intake during HS, refeeding after feed restriction increased circulating PRL in young mares (McManus & Fitzgerald 2000). Differing impacts of HS on circulating PRL have been reported, including no impact (Barb *et al.* 1991, Iguchi *et al.* 2012, An *et al.* 2020). Interestingly, PRL secretion can be influenced by E_2_ (Sosa *et al.* 2012), although HS in males also increases circulating PRL (Vesic *et al.* 2021). Thus, increased PRL as a consequence of HS has the potential to augment fertility in the pig.

#### Progesterone

The pig depends on a functional corpus luteum (CL) as the P_4_ source (Geisert *et al.* 2015, Lonergan *et al.* 2016, Spencer *et al.* 2016), which is required for conceptus implantation. Low P_4_ levels are associated with underdeveloped concepti (Spencer *et al.* 2016) and exogenous P_4_ supplementation can improve reproductive outcomes (Martinat-Botte *et al.* 1995, van Leeuwen *et al.* 2011, Muro *et al.* 2021). Since LPS reduces CL size and function (Suzuki *et al.* 2001), resulting in reduced P_4_, there is potential for the CL to be an HS target. Heat stress increases the production of pro-inflammatory cytokines, including TNFα (Kluger *et al.* 1997) and cultured bovine luteal cells exposed to TNFα had increased PGF_2α_ (Townson & Pate 1996). Short-term *in vitro* culture of bovine ovaries with constant LPS administration also increased PGF_2α_ concentration in the culture media (Lüttgenau *et al.* 2016).

Exposure to HS during the luteal phase of the estrous cycle resulted in reduced CL weight, suggesting either impact on cell viability or an alteration in the cellular composition of the CL (Bidne *et al.* 2019). However, the amount of P_4_ produced per milligram of CL tissue was increased by HS (Bidne *et al.* 2019), illustrating the steroidogenic impact of HS. Considering that early embryonic loss is a phenotypic hallmark of HS, reduced P_4_ production is a logical consequence of HS, and the CL may attempt to counter a reduction in CL tissue with increased steroid output. Importantly, supplementing a P_4_ analog restored CL weight during HS (Bidne *et al.* 2019). Whether the luteal cell types differ due to HS remains unknown, and the potential for P_4_ supplementation to augment HS-induced luteal insufficiency is conceivable.

#### Links between systemic impacts of HS

**Figure 2:**
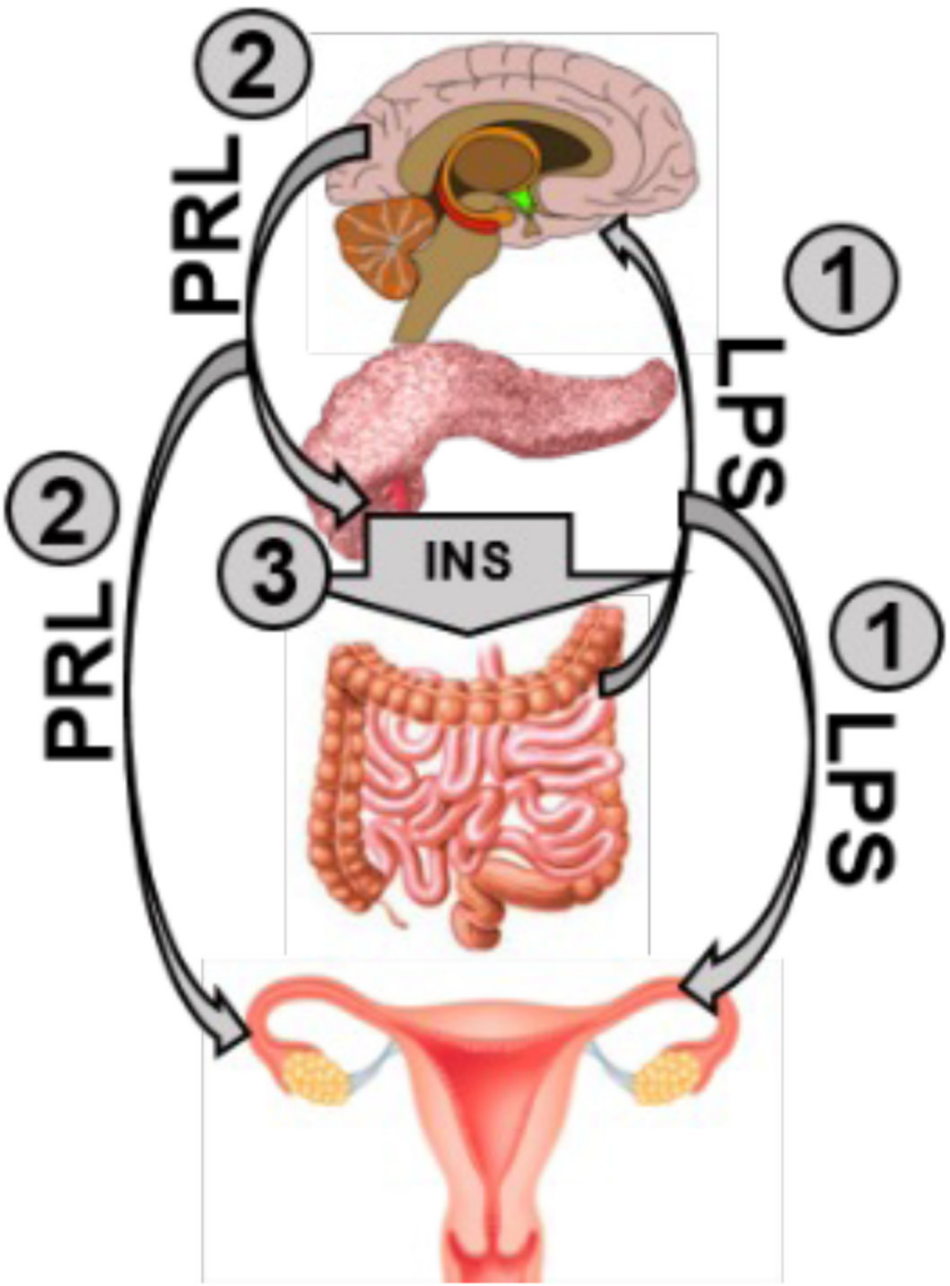
Potential links between HS-induced systemic alteration: (1) LPS infiltrates the system due to a leaky intestine, causing (2) PRL release from the anterior pituitary, which (3) results in pancreatic beta-cell proliferation and increased systemic insulin.

In non-porcine species, there are links between these systemic HS effects: LPS has a stimulatory effect on PRL secretion in anterior pituitary explants from sheep (Tomaszewska-Zaremba *et al.* 2018), and PRL can stimulate pancreatic beta-cell proliferation and insulin secretion (Ben-Jonathan *et al.* 2006, Arumugam *et al.* 2010) via the JAK/STAT pathway (Nielsen *et al.* 1992). Glucose-stimulated insulin secretion and insulin production can be induced via PRL in rat islet beta-cells during fetal (Møldrup *et al.* 1993, Freemark *et al.* 1995, Royster *et al.* 1995), neonatal (Brelje & Sorenson 1991, Brelje *et al.* 1994), and adult development (Møldrup *et al.* 1993). Furthermore, elevated PRL improves insulin sensitivity in males (Ruiz-Herrera *et al.* 2017). Interestingly, extra-pituitary PRL production from immune cells has been recently documented (Barrett *et al.* 2018) and enhances inflammatory signals caused by LPS exposure (Lopez-Rincon *et al.* 2013). Further, PRL blunts LPS-induced activation of inflammatory cytokines and TLR4/NF-κB signaling *in vitro* (Olmos-Ortiz *et al.* 2019, Flores-Espinosa *et al.* 2020), suggesting an anti-inflammatory role of PRL. Thus, during HS, compromised intestinal integrity can (1) leak intestinal contents into the systemic circulation, potentially inducing (2) PRL release from the pituitary, leading to (3) pancreatic beta-cell proliferation and insulin secretion. These alterations are summarized in Fig. 2.

### Intracellular ovarian signaling demonstrated to be altered during HS

**Figure 3:**
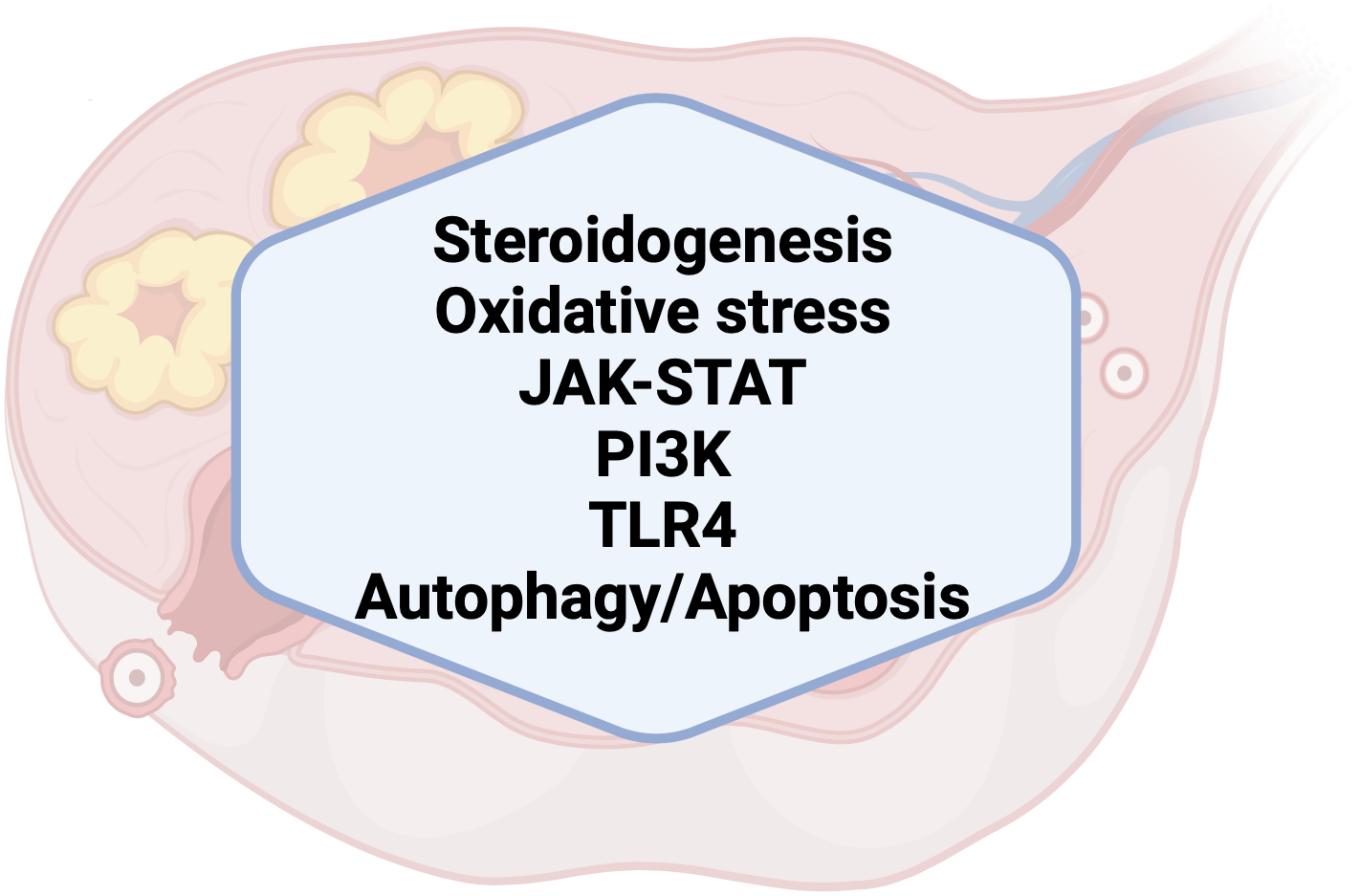
Summary of HS-induced ovarian molecular alterations. During HS, ovarian molecular pathways, including JAK–STAT, PI3K, oxidative stress, steroidogenesis, TLR4, and autophagy/apoptosis are functionally altered in ways that may hamper fertility.

The molecular pathways that are altered in the ovary as a consequence of HS are described below and summarized in Fig. 3. Several are linked to the systemic alterations *in vivo* during HS in mammals.

#### Insulin and phosphatidylinositol-3 kinase signaling

The ovary possesses insulin receptors (IR), heterodimers comprised of two alpha (α) and two beta (β) subunits (Sweet *et al.* 1987*a*,*b*, Kido *et al.* 2001). Insulin binds the α-subunits, activating the IR tyrosine kinase in the β-subunits, with subsequent auto-phosphorylation and recruitment of substrate adaptors, including the IR substrate (IRS 1–4) proteins. Defects in IRS, more specifically IRS1 and IRS2, cause female infertility (Poretsky *et al.* 1999, Brüning *et al.* 2000, Choi & Kim 2010). The impacts of HS on hyperinsulinemia and insulin-induced signaling in post-pubertal females who were estrus synchronized and experienced either TN or cyclical HS for 5 d during the follicular phase demonstrated increased ovarian IR abundance, emphasizing that hyperinsulinemia during HS alters ovarian insulin signaling (Dickson *et al.* 2018). Additionally, in pre-pubertal heat-stressed females, ovarian IR was increased (Nteeba *et al.* 2015) and mRNA encoding *IRS1* was elevated with increased pIRS1^Tyr632^, also supporting that ovarian insulin signaling is magnified by HS (Nteeba *et al.* 2015).

Increased PI3K activation during HS occurs in females (Nteeba *et al.* 2015). The importance of the PI3K signaling pathway on ovarian function is well established (Liu *et al.* 2006, 2007*a*,*b*, Reddy *et al.* 2008, 2009, Jagarlamudi *et al.* 2009, Adhikari *et al.* 2010). The viability of primordial follicles in the ovarian reserve and their activation into the growing follicular pool are maintained by the PI3K pathway (Reddy *et al.* 2005, Roskoski 2005, Liu *et al.* 2006, Jagarlamudi *et al.* 2009). The granulosa cell-produced growth factor, kit ligand, binds to its receptor c-KIT that is localized in the oocyte (Manova *et al.* 1990, Orr-Urtreger *et al.* 1990, Horie *et al.* 1991, Ismail *et al.* 1996), resulting in c-KIT phosphorylation and PI3K activation. The plasma membrane lipid phosphatidylinositol-4,5-bisphosphate is then transformed into phosphatidylinositol-3,4,5-trisphosphate and recruits lipid-binding domain proteins from the cytoplasm to the plasma membrane, where they are phosphorylated (Pawson & Nash 2000). One such protein is serine/threonine-protein kinase B (AKT) (Blume-Jensen & Hunter 2001, Cantley 2002, Fayard *et al.* 2005), which regulates a myriad of genes, including the forkhead box transcription factor (FOXO3) (Datta *et al.* 1999, Kim *et al.* 2000, Blume-Jensen & Hunter 2001, Nicholson & Anderson 2002, Manning & Cantley 2007). In the oocyte, FOXO3 regulates cell cycle arrest and apoptosis (Burgering & Medema 2003, Tran *et al.* 2003). When phosphorylated by pAKT, FOXO3 leaves the oocyte nucleus, resulting in oocyte activation and growth (Brunet *et al.* 2001). During HS, ovarian *AKT1* and *FOXO3* mRNA abundances are increased (Nteeba *et al.* 2015). In addition, HS increased pAKT1 protein, demonstrating that ovarian PI3K signaling is augmented by HS (Nteeba *et al.* 2015). The observed increased *FOXO3* may impair primordial follicle activation (Kops *et al.* 2002, Castrillon *et al.* 2003, Adhikari & Liu 2009), potentially contributing to adverse reproductive impacts of HS.

#### LPS/TLR4 signaling

Pigs subjected to cyclical HS during the follicular phase of the estrus cycle had increased ovarian TLR4 protein abundance, demonstrating that ovarian LPS signaling is active during HS. LPS binds to TLR4 to initiate a signal cascade culminating in NFκB activation by phosphorylation of subunit RELA and nuclear translocation (Poltorak *et al.* 1998, Hoshino *et al.* 1999). Activated NFκB upregulates pro-inflammatory cytokine expression (Akira *et al.* 2006, Lu *et al.* 2008) and excess cytokine production is detrimental to fertility (Adashi *et al.* 1989, Ghersevich *et al.* 2001, Herath *et al.* 2007). HS increased pRELA, the activated subunit of NFκB, in the pig ovary. Thus, during HS in post-pubertal females, the abundance of both ovarian TLR4 and pRELA is increased, supporting that the ovary is responsive to HS-induced endotoxemia.

#### Steroidogenesis

In granulosa cells from mice, HS reduced E_2_ and P_4_ along with decreased mRNA abundance of genes encoding steroidogenic proteins (Luo *et al.* 2016). In pre-pubertal heat-stressed female pigs, both ovarian STAR and CYP19A were dramatically increased by HS (Nteeba *et al.* 2015). Since these enzymes catalyze the initiating and concluding steps in E_2_ synthesis, the potential for HS to alter ovarian E_2_ production is probable. Contrarily, luteal protein abundance of steroid acute regulatory protein, 3 beta-hydroxysteroid, or prostaglandin F2α receptor were not different in the CL of pigs who experienced HS only during the luteal phase, despite elevated P_4_ per mg of CL tissue (Bidne *et al.* 2019). Thus, HS alters the ovarian abundance of proteins involved in steroid hormone synthesis, a critical aspect of female reproductive function.

#### JAK–STAT proteins

As mentioned above, the JAK/STAT pathway is activated by PRL. In pigs who experienced HS during the follicular phase of the estrous cycle, ovarian pSTAT5α/βTyr694/699 was increased, and during the luteal phase, HS increased STAT3 in the CL, potentially indicating that HS augments ovarian JAK–STAT signaling (Roach *et al.* 2022). This provides a molecular link between increased systemic PRL and altered ovarian PRL-induced intracellular signaling.

#### Heat shock proteins

Mammalian cells constitutively express certain HSP (even in the absence of stress, and these are known as cognate HSP), which are involved in protein folding, transfer of proteins across membranes, and developmental processes such as embryogenesis (Burel *et al.* 1992). Inducible HSP is produced in response to insults such as thermal or oxidative stress, ischemia, and bacterial or viral infections (Burel *et al.* 1992, Neuer 2000). Thus, HSPs are involved in cellular pathways responsive to both LPS and HS. Members of the HSPs have been noted to be responsive to a heat load in both mice (Bei *et al.* 2020) and to heat shock in pigs (Sirotkin & Bauer 2011). The chaperone proteins HSPA1A, HYOU1, and HSPE1 are decreased in response to HS, with a bidirectional effect on HSPH1. Heat shock protein family A (HSP70) member 1A (HSPA1A) and E member 1 (HSPE1 or HSP10) are chaperonins that assist in mitochondrial protein refolding (Hartman *et al.* 1992, Bakthisaran *et al.* 2015) during stress. Elevated ovarian HSPA1A abundance has been observed in estrous-synchronized pigs that were exposed to HS for five days during the follicular phase of the estrous cycle (Seibert *et al.* 2019), indicating HSPA1A as a potential chaperone biomarker in ovarian tissue during thermal stress regardless of reproductive developmental status. *HSP70* presence in granulosa cells and oocytes in pig ovaries has been determined (Li *et al.* 2017), suggesting that damage to oocyte and granulosa cell proteins by HS may ensue, potentially impairing follicle development and oocyte growth prior to puberty. Interestingly, heat shock protein family H (HSP110) member 1 (HSPH1) was increased by HS ovarian abundance. Thus, a robust HSP response to HS in the pig ovary is documented, potentially reflective of basal HSP’s functions to cope with cellular insults due to HS.

### Cell death pathways

Cellular homeostasis involves the removal of damaged cellular materials through the process of autophagy (Ryter *et al.* 2013), which involves several steps, the regulation of which can be post-translational (Mizushima 2007, Mizushima *et al.* 2011). Changes to the cellular morphology of follicles due to HS have been reported with increased vacuolization in both the oocyte and granulosa cells (Hale *et al.* 2017). Additionally, HS increased the protein abundance of beclin 1 (BECN1) and microtubule-associated protein 1 light chain 3 beta (LC3B-II), and autophagy-related gene 12 (ATG12) was decreased in abundance (Hale *et al.* 2017). Phosphorylated B-cell lymphoma 2 (BCL2) was increased by HS, and B-cell lymphoma 2 like 1 protein (BCL2L1) was also increased in the oocyte of prophase I-arrested oocytes (Hale *et al.* 2017). In cultured pig oocytes, HS increased the abundance of the ATG12–ATG5 complex and enhanced LC3B-II loss in oocytes (Hale *et al.* 2021). These findings suggest that ovarian pathways related to cell death and damaged cellular component turnover are altered by HS.

### Proteomic impacts of HS

A transcriptional profiling approach identified changes to mRNAs that were altered due to heat in cumulus–oocyte complexes of pigs and identified alterations to mRNAs involved in the HSP response and extracellular matrix (Liu *et al.* 2021). An untargeted approach using LC–MS/MS identified HS targets in the ovary of pre-pubertal gilts in the absence of the ovarian hormonal milieu (Roach *et al.* 2024*a*) discovered 178 ovarian proteins (76 increased and 102 decreased) that were impacted by HS. Compared to thermal neutral pigs on the same plane of nutrition, a greater proteomic response to HS was observed. Bioinformatic analysis identified 79 proteins to be consistently altered by HS, regardless of whether the profile was compared to thermal neutral fed or limit fed thermal neutral controls *ad libitum*. HS reduced the hypoxia upregulated 1 (HYOU1) protein (Roach *et al.* 2024*a*), potentially indicating that the ovary is experiencing hypoxic conditions in alignment with the alteration to blood flow during HS. Additional ovarian oxidative stress-related proteins, peroxiredoxin, thioredoxin-like 1, and glutathione *S*-transferase were also impacted (Roach *et al.* 2024*a*). Oxidative stress due to HS is reported (Adachi *et al.* 2009) and mitigation by antioxidant treatment improved the oocyte response to HS (Cavallari *et al.* 2019).

Immunological responsive proteins that were altered by HS included increased heterogeneous nuclear ribonucleoprotein L-like, complement C8 alpha chain, complement C2, histidine-rich glycoprotein, Galectin-10, and decreased Ficolin-2 (FCN2) (Roach *et al.* 2024*a*). FCN2 is an innate immune pattern recognition protein within the complement system (Garred *et al.* 2010). Combined with an increased abundance of the other identified immune response proteins during HS, this suggests immune activation is occurring within the ovary. There were also changes to heat shock protein family A (HSP70) member 1A (HSPA1A) and E member 1 (HSPE1 or HSP10) in response to HS (Roach *et al.* 2024*a*), complementing the targeted experimental approach discoveries already mentioned. Interestingly, HSP family H (HSP110) member 1 (HSPH1) was bidirectionally altered, with increased ovarian abundance in the HS compared with ad libitum TN gilts but decreased levels when compared to pair-fed thermal neutral gilts (Roach *et al.* 2024*a*). The physiological functions of the proteins affected by HS were chaperones, immunity, extracellular matrix, metabolism, and cell signaling (Roach *et al.* 2024*a*). These findings are in alignment with the systemic observations that occur at the phenotypic and endocrinological level in the HS female.

## Conclusion or future perspective

Infertility results as a consequence of HS and several systemic and ovary-specific alterations that are a consequence of an increased thermal load have been identified. Intestinal integrity failure as a consequence of re-routed blood flow *in vivo* is a credible crux for the subsequent physiological sequelae, and this concept is summarized in Fig. 2. The detrimental effects of LPS on ovarian function are documented; however, LPS is a single constituent of the intestinal tract and has been used experimentally as a proxy tool for the induction of endotoxemia. More detailed knowledge of the impacts of additional intestinal contaminants on the endogenous system is required in the context of HS. Whether improved intestinal integrity could mitigate HS-induced infertility is also an area for exploration. Altered blood flow during HS may itself be consequential for ovarian function, considering the necessity for nutrients and hormones within the female gonad. This concept is reinforced by findings of altered blood flow to the uterus (Nanas *et al.* 2021) and dominant follicles (Honig *et al.* 2016) during HS in non-porcine species. As addressed in this review, off feed during HS is detrimental to reproduction and specifically, ovarian function. Perhaps stimulation of appetite during HS could be a strategy to overcome this reduction in feed intake. There are endocrinological responses to HS that could be altered using pharmacological interventions, and these should be conducted singly to firmly establish firmly the *a priori* hormonal change in response to HS. Pre-emptively, however, is the determination of the temporal pattern of each physiological change to firmly identify the nexus of the HS response. A caveat to each of these suggested potential mitigation approaches is that the physiological response to a heat load is a logical attempt to prioritize the survival of the animal, and manipulation of any of these could be detrimental to animal health. Finally, the findings described in this review are focused on work from the porcine model, and investigating the physiological and molecular response to HS systemically and within the ovary at differing developmental stages is warranted.

## Declaration of interest

The authors declare that there is no conflict of interest that could be perceived as prejudicing the impartiality of the study reported. AFK is an Associate Editor of *Reproduction*. AFK was not involved in the review or editorial process for this paper, on which she is listed as an author.

## Funding

This work was funded through the Iowa State Universityhttp://dx.doi.org/10.13039/100009227 incentive program.

## Author contribution statement

AFK drafted and edited the manuscript; JKR and LHB contributed to writing and editing.
